# Brain-inspired predictive visual attention for spatiotemporal semantic understanding of complex film and television videos

**DOI:** 10.3389/fnbot.2026.1934032

**Published:** 2026-10-22

**Authors:** Peng Han

**Affiliations:** Nanjing Institute of Technology, Nanjing, Jiangsu, China

**Keywords:** brain-inspired visual attention, movie semantic understanding, predictive coding, spatio-temporal representation, video saliency detection

## Abstract

Due to the unique characteristics of film and television, such as montage editing and narrative structure jumps, semantically salient targets frequently experience temporal feature drift, making it difficult for existing models to maintain stable semantic understanding output when keyframes are lost. Inspired by predictive coding mechanisms in neuroscience, this paper proposes a spatiotemporal joint representation framework driven by brain-like visual attention. A pixel-level saliency heatmap is constructed using eye-tracking data as a physiological-level supervisory signal. A Dual-Stream Spatio-Temporal Encoding Network (DS-STEN) is designed: the spatial stream uses ResNet to extract single-frame salient features, while the temporal stream uses 3D convolution to model the optical flow field and capture cross-frame trajectories. A Kalman filter-driven predictive attention module is introduced into the fusion layer to estimate the target’s position and feature distribution in the next frame. The prediction results are injected into the temporal stream as *a priori* mask to actively correct abnormal responses during drift. The corrected spatiotemporal features are aggregated with semantic context through a Transformer cross-frame self-attention layer and input into a semantic classification head to generate scene-level and event-level labels. End-to-end joint training is performed using a weighted loss of saliency prediction, trajectory consistency, and semantic classification. Experiments show that the saliency prediction correlation coefficient (CC) reaches 0.846 and the normalized scanpath saliency (NSS) reaches 2.47 on the AIM2024 benchmark. In the TVQA+ dataset target tracking task, the cross-frame tracking accuracy in unoccluded scenes reaches 95.3%. In the MovieNet dataset scene semantic classification task, the average accuracy for Dialogue scenes reaches 93.5%. This paper provides a new spatiotemporal modeling paradigm with a neuroscience basis for semantic understanding of complex film and television videos.

## 1 Introduction

As the most important visual information carrier in contemporary times, film and television videos carry complex narrative structures and visual semantic information. With the continuous expansion of streaming media, the demand for automated semantic understanding of film and television content in fields such as intelligent retrieval and plot analysis is becoming increasingly urgent (Cho et al., 2026; You et al., 2024). Due to the unique characteristics of film and television videos such as montage editing and narrative jumps, semantically salient targets frequently experience feature temporal drift. Existing models are prone to semantic output collapse when keyframes are lost. Solving this problem is of great practical significance for promoting the intelligent processing of film and television content (Wang et al., 2023; Zhang et al., 2023b).

The field of video semantic understanding has accumulated a wealth of research results and public benchmarks. MovieNet, with its multimodal annotations covering 1,100 movies, provides systematic support for scene segmentation and character understanding (Chen et al., 2024; Ma et al., 2026); TVQA+ introduces frame-level target bounding box annotations in video question answering tasks, promoting joint research on visual localization and language alignment (Lei et al., 2020; Liang et al., 2024); and the AIM2024 Video Saliency Challenge gathers large-scale eye-tracking data, providing a physiological-level evaluation benchmark for brain-like visual attention modeling (Moskalenko et al., 2024; Zhu et al., 2025). The establishment of these benchmarks indicates that film and television video semantic understanding has become an important frontier direction in computer vision.

Existing methods exhibit limitations in three aspects of video understanding. In terms of video saliency prediction, spatiotemporal coding-based methods assume inter-frame smoothness, leading to systematic biases in prediction when faced with abrupt changes in gaze distribution at video editing points (Li and Liu, 2025; Zhang et al., 2023a). Regarding cross-frame target tracking, methods based on Siamese networks and correlation filtering rely on continuous updates to appearance templates; when occlusion or sudden changes in lighting cause template failure, the tracking link breaks down and lacks an active recovery mechanism (J. Liu et al., 2024a; Wei et al., 2022). In terms of semantic understanding, multimodal fusion methods based on Transformer lack physiological constraints on visual saliency, resulting in a lack of stable attention anchors for semantic feature aggregation in narrative jump scenarios (An et al., 2023; Zheng et al., 2024). None of these three methods deeply couple the predictive mechanism of brain-like visual attention with spatiotemporal representation, and the problem of temporal drift of salient targets remains unresolved.

To address the aforementioned issues, this paper, inspired by predictive coding mechanisms in neuroscience, constructs a brain-like visual attention-driven spatiotemporal joint representation framework for complex film and television videos. Gaussian kernel density estimation is used to transform eye-tracking fixation points into pixel-level salient heatmaps, with specific corrections applied to abrupt changes at editing points. A dual-stream spatiotemporal coding network, DS-STEN, is designed. The spatial stream uses ResNet to extract frame-by-frame salient semantic features, while the temporal stream uses 3D convolution to model the optical flow field and capture abrupt inter-frame motion changes. A Kalman filter-driven predictive attention module is introduced into the fusion layer, mapping the target state estimate to *a priori* attention mask for the next frame, actively correcting abnormal responses during drift. The corrected features are then aggregated across frames using a Transformer self-attention layer to generate narrative context, which is input into a semantic classification head to generate scene-level and event-level labels. This is then jointly trained end-to-end using a three-factor weighted loss. The main contributions of this paper are as follows: ① A pixel-level saliency heatmap construction method using eye-tracking data as physiological-level supervision signals is proposed, providing a neuroscience basis for brain-like attention modeling; ② A Kalman filter-driven predictive attention module is designed, unifying target state prediction and attention mask generation into a single framework; ③ A three-task joint training strategy is constructed to achieve synergistic optimization of brain-like attention, spatiotemporal representation, and semantic understanding.

## 2 Spatiotemporal representation method based on brain-inspired attention

### 2.1 Construction of pixel-level saliency heatmap

The distribution of observer gaze points changes abruptly before and after the editing point of a film or video shot. Directly constructing a heatmap using the original gaze coordinates will introduce discontinuous noise at the editing boundary, contaminating the monitoring signal of the subsequent network.

Let the eye-tracking fixation point set of the t -th frame be ${\left\{(x_{i}^{t},y_{i}^{t})\right\}}_{i=1}^{N_{t}}$ , where $N_{t}$ denotes the number of valid fixation points in this frame. To transform the discrete fixation points into a continuous spatial distribution, Gaussian kernel density estimation (Wang et al., 2021) is applied to construct the saliency response field for each frame. The saliency value at position (x,y) is defined as:

$$S_{t}(x,y)=\frac{1}{N_{t}}\sum_{i=1}^{N_{t}}\frac{1}{2\pi h^{2}}\exp(-\frac{{\left(x-x_{i}^{t}\right)}^{2}+{\left(y-y_{i}^{t}\right)}^{2}}{2h^{2}})$$ (1)

In Equation 1, h represents the kernel bandwidth parameter, determined based on video resolution and average gaze diffusion radius, controlling the spatial diffusion of the attention response. This estimation diffuses the influence of each gaze point to surrounding pixels in Gaussian form, outputting a continuous probability density field as the initial frame-level saliency response.

Because there is a statistical discontinuity in the gaze distribution of adjacent frames at the clipping point, cross-frame aggregation will lead to blurred heatmap boundaries. Therefore, a frame-difference-based clip detection mechanism (Lu and Shi, 2013; Yuan and Zhang, 2023) is introduced, where the clip positions are determined by comparing the mean grayscale difference between adjacent frames $\Delta_{t}$ with an adaptive threshold τ :

$$c_{t}=1[\Delta_{t}>\tau ],\Delta_{t}=\frac{1}{\mathrm{HW}}\sum_{x,y}|I_{t}(x,y)-I_{t-1}(x,y)|$$ (2)

In Equation 2, H and W denote the height and width of the frame, respectively, $I_{t}(x,y)$ represents the grayscale value at position (x,y) in the t -th frame, and $c_{t}\in \{0,1\}$ denotes the clip marker. When $c_{t}=1$ , the heatmap temporal aggregation chain is interrupted at this frame to prevent the mixed propagation of attention distributions across shot boundaries.

Under the constraint of the clip marker, the frame-level saliency responses of consecutive frames within the same shot are weighted and fused using the temporal decay weight $w_{k}=\exp(-\lambda |t-k|)$ , obtaining the final pixel-level saliency heatmap ${\hat{S}}_{t}(x,y)$ :

$${\hat{S}}_{t}(x,y)=\frac{\sum_{k\in T_{t}}w_{k}S_{k}(x,y)}{\sum_{k\in T_{t}}w_{k}}$$ (3)

In Equation 3, $T_{t}$ denotes the valid frame window centered on the t -th frame after truncation by the clip marker, and λ represents the decay coefficient, which suppresses the interference of distant frames on the saliency estimation of the current frame. The weighted fusion aggregates gaze consistency information within the same shot into a temporally stable saliency distribution. ${\hat{S}}_{t}(x,y)$ is mapped to the interval [0,1] through maximum normalization and is then used as a pixel-level physiological supervision signal for the loss computation layer of the subsequent two-stream encoding network.

### 2.2 Dual-stream spatiotemporal coding networks oriented to the characteristics of film and television editing

Single-frame spatial features cannot capture the information of sudden motion changes before and after the editing point in film and television, while the modeling of inter-frame optical flow by a single temporal stream lacks spatial positioning constraints on static semantic regions. The separation of these two types of information leads to semantic discontinuities in the fused representation at the editing boundary.

The spatial stream takes the original RGB image $I_{t}\in R^{H\times W\times 3}$ of the t -th frame as input and extracts spatial semantic features layer by layer through the ResNet backbone (Lin et al., 2023). To inject the physiological-level supervision information from the saliency heatmap ${\hat{S}}_{t}$ into the spatial features, ${\hat{S}}_{t}$ is used to spatially weight the final-layer feature map $F_{t}^{s}\in R^{H^{\prime }\times W^{\prime }\times C}$ , obtaining the saliency-enhanced spatial representation ${\tilde{F}}_{t}^{s}$ :

$${\tilde{F}}_{t}^{s}=F_{t}^{s}⊙\phi ({\hat{S}}_{t})$$ (4)

In Equation 4, ϕ(·) denotes the bilinear interpolation operation, which downsamples ${\hat{S}}_{t}$ from the original resolution to the spatial scale $H^{\prime }\times W^{\prime }$ consistent with $F_{t}^{s}$ , ⊙ denotes element-wise multiplication, and C represents the number of feature channels. ${\tilde{F}}_{t}^{s}$ suppresses redundant activations in non-attention regions through saliency weighting while preserving semantic responses consistent with human gaze distributions, thereby directly constraining the selection range of spatial features in subsequent fusion layers.

The temporal stream takes the optical flow field sequence ${\left\{O_{t}\right\}}_{t=1}^{T}$ composed of consecutive T frames as input, where $O_{t}\in R^{H\times W\times 2}$ represents the horizontal and vertical optical flow components of the t -th frame. To simultaneously model local motion textures and cross-frame motion variations, the temporal stream applies the 3D convolution kernel $K\in R^{C_{\text{out}}\times C_{\text{in}}\times d\times k\times k}$ to perform spatiotemporal joint convolution on the optical flow sequence. The update of the l -th layer temporal feature map $F^{\left(l\right)}$ is formulated as:

$$F^{\left(l\right)}=\sigma (K^{\left(l\right)}*F^{\left(l1\right)}+b^{\left(l\right)})$$ (5)

In Equation 5, ∗ denotes the 3D convolution operation, d represents the temporal kernel depth, k denotes the spatial kernel size, $b^{\left(l\right)}$ is the bias term, and σ(·) represents the ReLU activation function. The temporal kernel depth d spans the shot boundaries defined by the clip detection marker $c_{t}$ , enabling the 3D convolution to capture abrupt gradient variations of the optical flow field at clip points. The output temporal feature $F_{t}^{o}\in R^{H^{\prime }\times W^{\prime }\times C}$ encodes the inter-frame motion variation patterns before and after clip transitions (Hui et al., 2023; Yang et al., 2024).

The two-stream features ${\tilde{F}}_{t}^{s}$ and $F_{t}^{o}$ are concatenated along the channel dimension and compressed to a unified dimension through a 1×1 convolution, forming the joint spatiotemporal representation $F_{t}$ :

$$F_{t}=W_{f}\cdot [{\tilde{F}}_{t}^{s}\|F_{t}^{o}]+b_{f}$$ (6)

In Equation 6, $W_{f}\in R^{C\times 2C}$ denotes the channel fusion weight matrix, $b_{f}$ represents the bias term, and [·∥·] denotes the concatenation operation along the channel dimension. $F_{t}$ simultaneously incorporates spatial semantic localization information constrained by saliency and clip-aware temporal motion encoding, serving as the state observation input for the subsequent Kalman filtering predictive attention module and providing joint spatiotemporal priors for cross-frame estimation of target position and feature distribution.

To visually demonstrate the feature extraction and fusion process of the dual-stream spatiotemporal coding network, its overall structure is shown in Figure 1.

**Figure 1:**
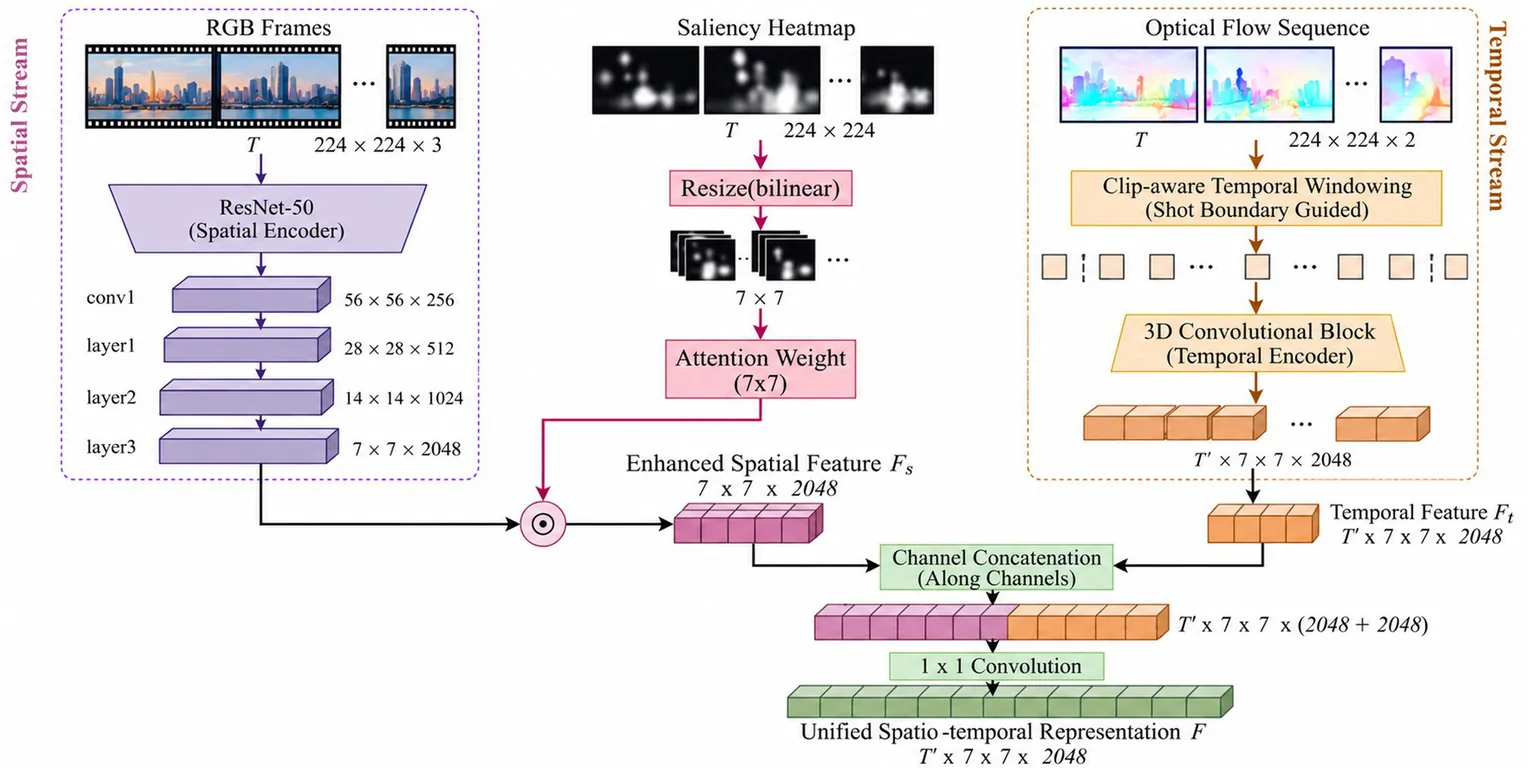
DS-STEN dual-stream spatiotemporal coding network structure. AI-assisted illustrative elements generated using OpenAI ChatGPT Images; see Supplementary material for prompts, tool details, and disclosure. The illustrative scenes are conceptual, not actual experimental video frames.

Figure 1 illustrates the overall data flow and feature fusion process of the dual-stream spatiotemporal coding network. The spatial stream takes RGB video frames as input, extracts spatial semantic features through ResNet, and combines them with pixel-level saliency heatmaps to enhance salient regions. The temporal stream takes optical flow sequences as input, extracts motion features from consecutive frames through 3D convolution to form a temporal representation. The two feature streams are fused through channel concatenation and 1 x 1 convolution to generate a unified joint spatiotemporal representation, providing a consistent feature representation for subsequent spatiotemporal information modeling.

### 2.3 Kalman filter-driven predictive attention module

The joint spatiotemporal representation $F_{t}$ only encodes the observation state of the current frame. When occlusion or sudden changes in light cause the target features to drift in the next frame, there is a lack of predictive constraints on the future position and feature distribution of the target. The temporal stream will passively accept abnormal feature responses and will not be able to actively correct them.

To map the current target state into a quantifiable state vector, $F_{t}$ is processed through global average pooling and linear projection to generate the target state vector $z_{t}\in R^{d}$ , where d denotes the dimensionality of the state space. Kalman filtering uses a linear dynamics assumption to perform temporal modeling of the target state (S. Liu et al., 2024b; Yan et al., 2024), and the state transition equation is:

$${\hat{z}}_{t+1|t}=Az_{t|t}+q_{t}$$ (7)

In Equation 7, $A\in R^{d\times d}$ denotes the state transition matrix, which encodes the linear motion prior of the target in the feature space. ${\hat{z}}_{t+1|t}$ represents the prior prediction of the t+1 -th frame based on the estimation of the t -th frame, and $q_{t}∼N(0,Q)$ denotes the process noise, where the covariance matrix Q characterizes the uncertainty of state transition. ${\hat{z}}_{t+1|t}$ takes the filtered estimation of the current frame as the starting point, extends the motion inertia of the target to the next frame, and forms a feature-level prediction prior.

When the observation $z_{t+1}^{\mathrm{obs}}$ of the t+1 -th frame arrives, the prior prediction and the actual observation are weighted and fused using the Kalman gain $K_{t+1}$ to obtain the posterior estimation:

$$z_{t+1|t+1}={\hat{z}}_{t+1|t}+K_{t+1}\;(z_{t+1}^{\mathrm{obs}}H{\hat{z}}_{t+1|t})$$ (8)

In Equation 8, $H\in R^{d\times d}$ denotes the observation matrix, which maps the state space into the observation space. $K_{t+1}$ is jointly determined by the predicted covariance and the observation noise covariance R , automatically increasing the weight of the prior prediction and suppressing interference from abnormal observations when target feature drift occurs. The posterior estimation $z_{t+1|t+1}$ integrates the motion prior and the current observation to form a corrected representation of the target’s true state (Ma et al., 2024; Zhang, 2025).

The prior prediction ${\hat{z}}_{t+1|t}$ carries the predicted information of the target position and feature distribution in the next frame. It is transformed through a transposed linear projection and spatial reshaping to recover a feature map with the same dimension as $F_{t}$ , and then passed through a Sigmoid activation function to generate the prior attention mask $M_{t+1}\in R^{H^{\prime }\times W^{\prime }\times C}$ . The generated mask is subsequently injected into the temporal flow to modulate the joint spatiotemporal representation of the t+1 -th frame:

$$F_{t+1}^{\mathrm{cor}}=F_{t+1}⊙M_{t+1}+F_{t+1}$$ (9)

In Equation 9, ⊙ denotes element-wise multiplication, the residual term $F_{t+1}$ preserves the complete information of the original spatiotemporal features to prevent excessive suppression, and $F_{t+1}^{\mathrm{cor}}$ represents the corrected spatiotemporal representation after mask modulation. The mask $M_{t+1}$ enhances feature responses in the target region centered on the predicted position while suppressing abnormal activations in drift regions, enabling the corrected representation to maintain stable anchoring to the actual target region even under abrupt target feature variations. The overall information flow of the predictive attention module is shown in Figure 2. The joint spatiotemporal features sequentially complete state representation, Kalman prediction update, predictive attention generation, and feature correction. The outputs of each stage maintain a continuous mapping relationship, forming a unified predictive attention modeling process.

**Figure 2:**
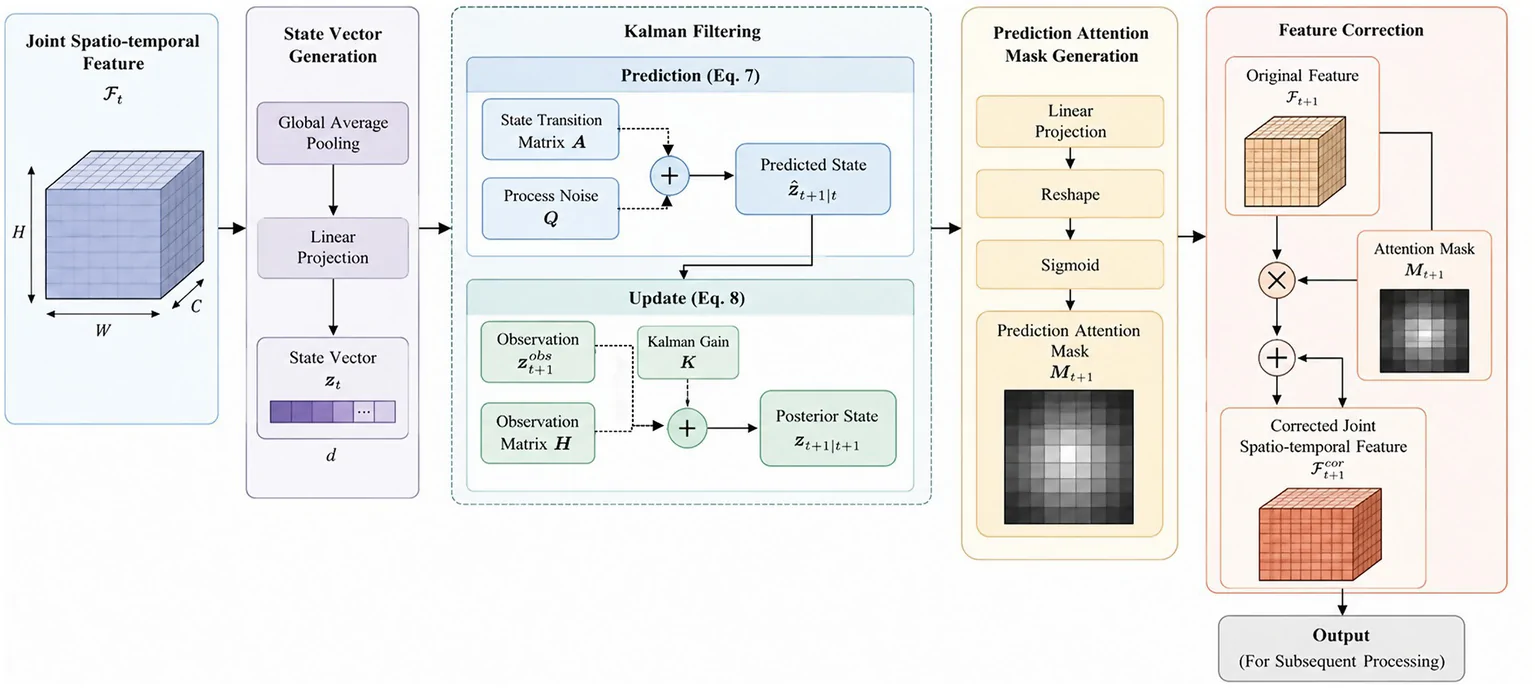
Kalman filter-driven predictive attention generation and feature modification process.

Figure 2 illustrates the internal computational flow of the predictive attention module. The joint spatiotemporal features are transformed into a state vector through global average pooling and linear mapping. State prediction and observation updates are fused within the Kalman filter framework. The posterior state is then transformed into a predictive attention mask through linear mapping, spatial reshaping, and Sigmoid activation. The mask is modulated element-wise with the joint spatiotemporal features and combined with residual connections to form a corrected joint spatiotemporal feature, providing a stable and consistent feature representation for subsequent modules.

$F_{t+1}^{\mathrm{cor}}$ is used as the downstream input of the posterior estimation $z_{t+1|t+1}$ and is transmitted to the Transformer cross-frame self-attention layer for multi-frame semantic context aggregation.

### 2.4 Cross-frame semantic understanding and joint training strategy

Although the modified spatiotemporal features suppress intra-frame drift noise, they are limited by the local field of view of a single frame and cannot capture semantic associations over long time spans. Furthermore, a single supervisory signal is insufficient to constrain the completeness of the feature space. Therefore, it is necessary to construct a cross-frame context aggregation mechanism and introduce multi-source supervisory signals to establish temporal consistency constraints (Bi et al., 2024; Wu et al., 2025). The corrected feature sequence of consecutive L frames ${\left\{F_{t}^{\mathrm{cor}}\right\}}_{t=1}^{L}$ is injected with temporal positional information through positional encoding and then fed into the Transformer encoder. The multi-head self-attention mechanism (Alfasly et al., 2022) is applied to compute the correlation weight matrix $A_{t}$ among frame-level features, thereby capturing long-range dependencies across shot boundaries:

$$A_{t}=\text{softmax}(\frac{Q_{t}K_{t}^{⊤}}{\sqrt{d_{k}}})$$ (10)

In Equation 10, $Q_{t}$ and $K_{t}$ are the query and key vectors obtained through linear projection of the input features, respectively, $d_{k}$ is the scaling factor of the vector dimension, and the weight matrix $A_{t}$ quantifies the dependency strength between the current frame and other frame features. After Softmax normalization, the value vectors $V_{t}$ are weighted and aggregated to obtain the enhanced feature ${\hat{F}}_{t}=A_{t}V_{t}$ , which explicitly encodes cross-frame temporal semantic dependencies and eliminates the uncertainty of single-frame features (Liang et al., 2023; Sun et al., 2024). After global average pooling compresses the spatial dimension of the enhanced feature ${\hat{F}}_{t}$ , it is fed into the fully connected layer to map into the semantic label space. The prediction probability distribution of scenes and event categories is obtained through Softmax normalization as $P(y_{t}|{\hat{F}}_{t})$ :

$$P(y_{t}|{\hat{F}}_{t})=\frac{\exp(W_{c}\cdot \mathrm{GAP}({\hat{F}}_{t})+b_{c})}{\sum_{j=1}^{C}\exp{\left(W_{c}\cdot \mathrm{GAP}({\hat{F}}_{t})+b_{c}\right)}_{j}}$$ (11)

In Equation 11, $W_{c}$ and $b_{c}$ represent the classification layer weights and biases, respectively, and C represents the total number of semantic categories. This probability output realizes the mapping from low-level features to high-level semantic labels. To ensure the coordinated optimization of spatiotemporal representation, trajectory prediction, and semantic understanding tasks, a multi-task joint loss function $L_{\text{total}}$ is constructed, which includes saliency prediction, trajectory consistency, and semantic classification. The network parameters are optimized using gradient anti-orthogonal optimization.

$$\begin{array}{l} L_{\text{total}}=\alpha D_{\mathrm{KL}}({\hat{S}}_{t}∥S_{t}^{\mathrm{gt}})+\beta ∥{\hat{z}}_{t+1|t}\\ -z_{t+1}^{\mathrm{obs}}{∥}^{2}-\gamma \sum_{c=1}^{C}y_{c}\log P(y_{c}|{\hat{F}}_{t}) \end{array}$$ (12)

In Equation 12, $D_{\mathrm{KL}}$ denotes the Kullback–Leibler divergence used to measure the distribution discrepancy between the predicted heatmap ${\hat{S}}_{t}$ and the ground-truth heatmap $S_{t}^{\mathrm{gt}}$ . The second term represents the Euclidean distance loss between the predicted state and the observed state, while the third term is the semantic classification cross-entropy loss. $y_{c}$ denotes the ground-truth label, and α , β , and γ are weighting coefficients. This joint loss constrains the model to maintain feature trajectory continuity under physiological-level attention constraints and output stable classification results at the semantic level.

## 3 Experimental setup for semantic understanding of film and television videos

### 3.1 Dataset and evaluation metrics

The AIM2024 Visual Attention Benchmark Version V1.2 was obtained from publicly available academic databases and used to verify pixel-level saliency prediction accuracy. Data collection spanned from January to June 2023, covering subjects from multiple countries in North America, Europe, and Asia to ensure sample diversity of visual perception patterns. The total number of features included both RGB pixel features and optical flow motion features. Feature selection was based on saliency correlation analysis. Labels were derived from saliency heatmaps generated by Gaussian blurring of fixation point coordinates collected by eye trackers. All labeled data underwent secondary manual verification to ensure reliability. Missing frames were filled using linear interpolation, and abnormal fixations were identified and removed using the standard deviation threshold criterion. Reliability testing was performed using Cronbach’s alpha. Alpha coefficient screening was performed, and the sample sizes retained after each step were 560, 120 and 120, respectively. The dataset was divided into three subsets based on the source film type: action, drama, and science fiction. The training set, validation set, and test set were divided in a ratio of 7:1.5:1.5, and each category was evenly distributed in each set. All data involving human facial regions were blurred and desensitized to comply with ethical standards. The boundaries of this dataset are limited to the visual attention distribution within specific film and television materials. TVQA+ Multimodal Video Question Answering Benchmark Version V1.0 was obtained from a public dataset portal and is used to evaluate cross-frame target tracking and event understanding capabilities. The data collection spans from 2015 to 2018, covering TV series broadcast in English-speaking countries. The data coverage mainly includes mainstream film and television works. The total number of features includes video frame appearance features, optical flow features, and text semantic features. The labels are sourced from manually annotated target bounding boxes and event answers. The label generation process has undergone consistency checks by multiple people. A small amount of label noise exists and has been smoothed through a mode voting mechanism. The dataset is stratified by video content complexity, with a training set, validation set, and test set ratio of 7:1.5:1.5. Small target categories account for approximately 20% of the test set, indicating class imbalance. All information involving personal privacy has been de-identified. The boundaries of this dataset are limited to the audiovisual semantic associations within specific TV series. The MovieNet Movie Scene Analysis Dataset, version V1.0, was obtained from a publicly available academic platform and used to validate the performance of scene-level semantic classification. The data collection spans films released between 1900 and 2020, covering various film genres from the global film industry. The total number of features includes scene visual features and metadata features. Labels are generated from film scripts and manual review, and have undergone validity checks. Missing scenes were filled using frame-by-frame inference. The proportions and sample sizes of the training, validation, and test sets are shown in the table. There is an imbalance in the long-tail distribution of each category. The data has been authorized for use and sensitive copyright watermarks have been removed. The boundaries of this dataset are limited by the era and style distribution of the selected film library. Details of the sample size distribution for the three datasets are shown in Table 1.

**Table 1:** Details of dataset sample size distribution.

Dataset	Total sample size	Training set	Validation set	Test set	Category/Condition breakdown
AIM2024	800	560	120	120	Action: 280; Drama: 260; Sci-Fi: 260
TVQA+	15,000	10,500	2,250	2,250	Normal lighting: 9000; Low lighting and shading: 6000
MovieNet	500	350	50	100	—

The selection of evaluation metrics should consider both overall performance fit and robustness under class imbalance conditions to avoid evaluation bias caused by a single metric. The Pearson correlation coefficient is used to measure the linear correlation between the predicted saliency heatmap and the actual heatmap, and the calculation formula is as follows:

$$\mathrm{CC}=\frac{\sum_{i}(S_{i}-\overset{`}{S})({\hat{S}}_{i}-\overset{`}{\hat{S}})}{\sqrt{\sum_{i}{\left(S_{i}-\overset{`}{S}\right)}^{2}}\sqrt{\sum_{i}{\left({\hat{S}}_{i}-\overset{`}{\hat{S}}\right)}^{2}}}$$ (13)

In Formula 13, $S_{i}$ and ${\hat{S}}_{i}$ represent the true value and predicted value, respectively, $\overset{`}{S}$ and $\overset{`}{\hat{S}}$ represent the corresponding means. This indicator reflects the overall consistency between the model’s predicted distribution and the human gaze distribution, and is suitable for evaluating the ability to model global saliency, but it is not sensitive to local extrema. Normalized scan path saliency is used to evaluate the saliency value of the prediction at a fixed gaze point, and the calculation formula is as follows:

$$\mathrm{NSS}=\frac{1}{N}\sum_{i=1}^{N}\frac{\hat{S}(x_{i},y_{i})-\mu_{\hat{S}}}{\sigma_{\hat{S}}}$$ (14)

In Formula 14, $\left(x_{i},y_{i}\right)$ is the coordinate of the i -th fixation point, $\mu_{\hat{S}}$ and $\sigma_{\hat{S}}$ are the mean and standard deviation of the prediction map. This metric captures the model’s ability to highlight key regions by focusing on the prediction intensity of the actual fixated area, complementing CC. The mean average precision (mAP) is used to measure the overall performance of multi-class scene classification tasks, and its calculation formula is as follows:

$$\mathrm{mAP}=\frac{1}{Q}\sum_{q=1}^{Q}\mathrm{AP}(q)$$ (15)

In Formula 15, Q represents the total number of categories, and AP(q) represents the average precision of the q category. This metric, calculated by averaging across all categories, effectively mitigates evaluation bias caused by class imbalance and objectively reflects the model’s generalization performance under long-tailed distributions. The intersection-over-union ratio (IoU) measures the overlap between predicted and ground truth bounding boxes during cross-frame tracking, and its calculation formula is as follows:

$$\mathrm{IoU}=\frac{\text{Area}(B_{p}\cap B_{\mathrm{gt}})}{\text{Area}(B_{p}\cup B_{\mathrm{gt}})}$$ (16)

In Formula 16, $B_{p}$ and $B_{\mathrm{gt}}$ represent the predicted bounding box and the true bounding box, respectively. This metric quantifies the localization accuracy and provides a more stringent geometric constraint evaluation than simple accuracy in occlusion and drift scenarios, ensuring the spatial compactness of the tracking results.

### 3.2 Experimental environment and model configuration

The experiment was conducted on a high-performance server platform equipped with an Intel Xeon Gold 6248R processor and 256 GB of DDR4 ECC memory. Model training was performed using a parallel computing environment consisting of four NVIDIA GeForce RTX 4090 GPUs, each with 24 GB of VRAM to accommodate the large-scale feature maps of the dual-stream network. Ubuntu 20.04 LTS was chosen as the operating system to ensure stability and compatibility. PyTorch 2.0.1 was used as the deep learning framework to support dynamic computation graph construction and efficient tensor operations. The computational backend relied on CUDA 11.8 and cuDNN 8.6 acceleration libraries, and Python 3.9 was used as the programming language. During data preprocessing, all input video frames were uniformly scaled to 256 px resolution and cropped to 224 px to adapt to the network input. The TV-L1 algorithm was used to extract the optical flow field as the temporal input. Simultaneously, enhancement strategies such as random horizontal flipping, random cropping, and color dithering were introduced to simulate the changes in perspective and lighting during film shooting. The spatial-stream network uses a ResNet-50 pre-trained on the ImageNet dataset as its backbone, leveraging transfer learning to accelerate model convergence. The temporal-stream network employs 3D convolutional kernels with a kernel size of 3 × 3 × 3 to capture local spatiotemporal motion features. The Kalman filter-driven predictive attention module sets the state vector dimension to 512, and initializes the process noise covariance and observation noise covariance to 0.01 and 0.1, respectively, to balance motion priors and observation confidence. The Transformer cross-frame self-attention layer contains 4 encoder layers and 8 attention heads to fully aggregate long-term temporal semantic context. The training optimization process uses the AdamW optimizer, setting the initial learning rate to 1 × 10^−4^ and the weight decay coefficient to 1 × 10^−4^. A linear warmup strategy is applied in the first 5 epochs to stabilize gradient updates, followed by a cosine annealing learning rate scheduler to dynamically adjust the learning rate over 60 training epochs. The weight coefficients for saliency prediction, trajectory consistency, and semantic classification in the joint loss function are set to 1.0, 0.5, and 1.0, respectively, to balance the multi-task learning objectives in end-to-end training. Detailed hyperparameter configurations and training strategies for each module are summarized in Table 2.

**Table 2:** Details of model hyperparameter configuration and training strategy.

Module categories	Parameter name	Numerical settings
Hardware environment	GPU model and video memory	NVIDIA RTX 4090/24GB
Input preprocessing	Resolution cropping and optical flow algorithms	224×224 /TV-L1
Spatial stream network	Backbone network and pre-trained dataset	ResNet-50/ImageNet
Temporal stream network	3D convolution kernel size	3×3×3
Predictive attention module	State vector dimension	512
Predictive attention module	Process noise and observation noise	0.01/0.1
Transformer module	Number of encoder layers and number of attention heads	4/8
Optimization strategy	Optimizer and Weight Decay	AdamW/ 1×10−4
Training strategy	Initial learning rate and Warmup rounds	1×10^−4^/5
Training strategy	Total number of training rounds and batch size	60/32
Loss function weights	α,β,γ	1.0/0.5/1.0

### 3.3 Comparison methods and ablation experiment design

The selection of baseline models for comparison covers traditional computational models, classic deep learning architectures, and current cutting-edge methods to ensure the comprehensiveness and gradient of the evaluation. The Itti-Koch model, as a traditional benchmark, uses a center-periphery difference operator to simulate low-level biological visual features. However, it relies on static features such as color, intensity, and orientation and cannot capture high-level semantics or dynamic temporal context, thus establishing a low-level performance baseline. SALICON represents a classic deep learning method, treating saliency prediction as a regression problem through deep convolutional networks. While significantly improving feature representation capabilities, it primarily focuses on static image frames and lacks dedicated temporal modeling mechanisms for complex motions and editing in videos. TASED-Net, as an advanced 3D convolutional architecture focused on temporal saliency, uses dilated 3D convolutions in its encoder-decoder structure to capture long-distance temporal dependencies. Its difference lies in its purely data-driven convolutional feature extraction, lacking the brain-like predictive coding mechanism or biologically inspired Kalman filter-guided active correction used in the proposed framework. VideoMAE represents a cutting-edge approach to video representation learning using visual Transformers. It employs masked autoencoders for large-scale pre-training to obtain general video features. While it possesses powerful global representation capabilities, it lacks a dual-stream fusion architecture designed for film and television editing structures and also lacks explicit dynamic prediction modeling for target state drift.

The ablation experiment design aims to verify the contribution of key components to the overall performance, and the proposed complete model is denoted as M0. To evaluate the effectiveness of the Kalman filter-driven predictive attention module, variant M1 removes the predictive state estimation and prior attention mask injection mechanism, directly inputting the original temporal flow features into the semantic aggregation layer. This design aims to test the necessity of predictive coding in handling feature drift and anomalous observations. To analyze the role of the Transformer cross-frame self-attention layer, variant M2 replaces the Transformer encoder with multi-layer 3D convolution and global average pooling, verifying the advantage of global context aggregation in resolving long-distance semantic dependencies compared to local convolution operations. To evaluate the contribution of the joint training strategy, variant M3 retains the full network architecture but uses only the saliency prediction loss for single-task training, removing the trajectory consistency and semantic classification losses. To distinguish prediction-guided feature modulation from state prediction itself, variant M4 retains Kalman state prediction but removes the predictive attention mask injection mechanism. This scheme is used to evaluate the specific gains of multi-task learning in enhancing feature discriminative power and robustness. By comparing the above systematic variations, we can clarify the independent value of each design decision in complex film and television semantic understanding tasks.

## 4 Results analysis

### 4.1 Analysis of saliency prediction accuracy

This section analyzes the saliency prediction performance of the proposed DS-STEN model across different film and television genres on the AIM2024 dataset. It compares and evaluates DS-STEN with Itti-Koch, SALICON, TASED-Net, and VideoMAE on three subsets: action, drama, and science fiction. Itti-Koch represents the traditional visual saliency model, SALICON represents a convolutional network-based saliency prediction method, TASED-Net represents a video spatiotemporal saliency network, VideoMAE represents a visual Transformer-based video representation method, and DS-STEN is the proposed dual-stream spatiotemporal coding model. The evaluation uses the Pearson correlation coefficient (CC) to measure the consistency between the predicted saliency map and the actual gaze distribution, and the normalized scan path saliency (NSS) to evaluate the response intensity of the actual gaze region. The average performance of each model across different film and television genres is statistically analyzed under a unified experimental environment and the same training and testing partitioning conditions, as shown in Figure 3.

**Figure 3:**
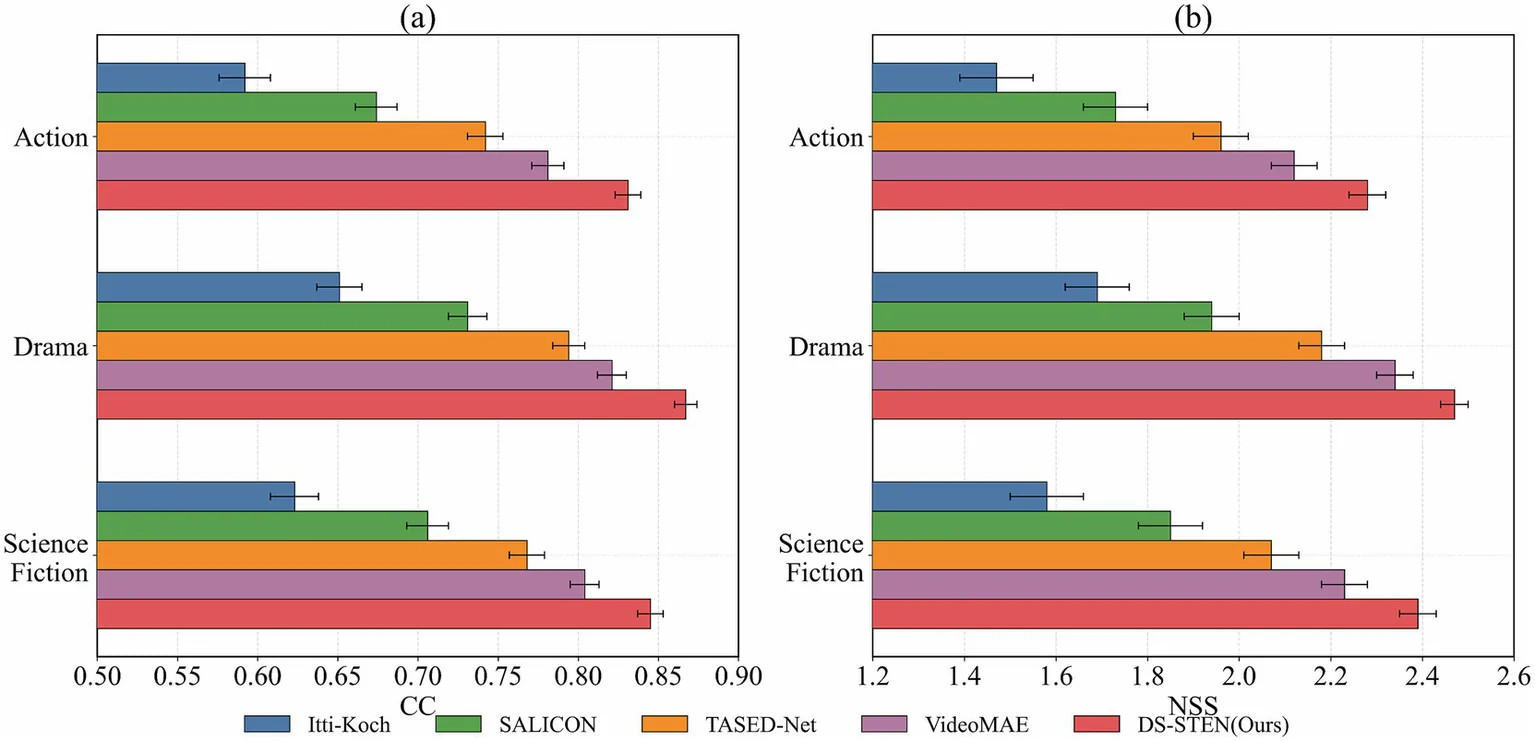
Comparison of saliency prediction performance of AIM2024 dataset under different film and television genres: **(a)** Comparison of CC index under different film and television genres; **(b)** Comparison of NSS index under different film and television genres.

Figure 3 shows that the saliency prediction accuracy of each model is generally higher in drama scenes than in action and science fiction scenes, reflecting that stable shots and continuous narratives reduce the difficulty of modeling visual attention distribution. DS-STEN achieved the highest performance in all three subsets: CC reached 0.831 in action scenes (a 0.050 improvement over VideoMAE), and NSS reached 2.28 (a 0.16 improvement); CC and NSS reached 0.867 and 2.47 in drama scenes, respectively; and CC reached 0.845 and NSS reached 2.39 in science fiction scenes. The performance gap between models was more significant in action scenes, indicating that high-speed motion, frequent editing, and target displacement have a stronger impact on saliency prediction. Dual-stream spatiotemporal coding and predictive attention mechanisms enhance the continuity of attention regions across frames, thereby maintaining high consistency in saliency distribution and response intensity in gaze regions. Overall, the results show that DS-STEN maintains stable and leading saliency prediction performance across different film and television genres.

### 4.2 Cross-frame target consistency tracking analysis

Target tracking in video is susceptible to temporal drift due to target occlusion and camera movement, which interferes with the continuity of semantic association across frames. To evaluate the impact of the predictive attention module on the ability to preserve target state, three typical scenarios—no occlusion, target occlusion, and camera movement—were set up on the TVQA+ dataset. Comparative experiments were conducted on Itti-Koch, SALICON, TASED-Net, VideoMAE, and the proposed method. Tracking Accuracy was used to evaluate the cross-frame target tracking accuracy in different scenarios, and Target Position Error was used to measure the offset distance between the target center position and the true position in consecutive frames. The effect of the predictive attention module on correcting target temporal drift was analyzed from the perspectives of overall performance and temporal variation. The results are shown in Figure 4.

**Figure 4:**
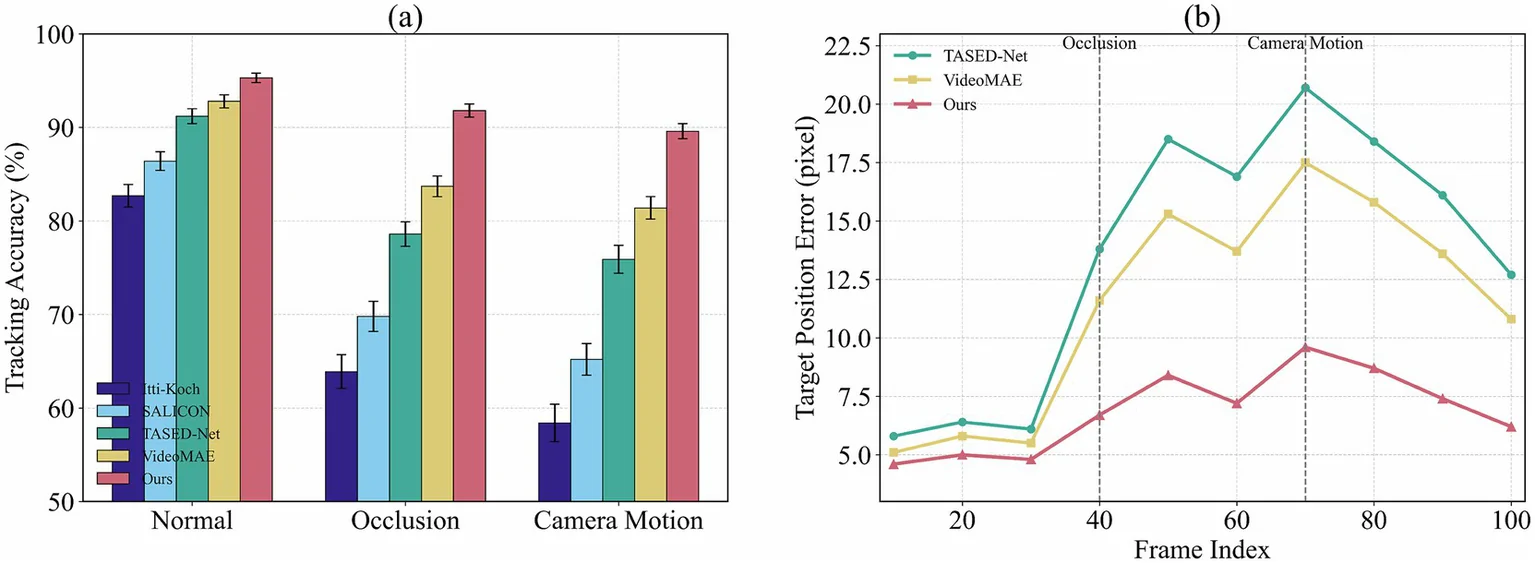
Cross-frame target consistency analysis of the predictive attention module. **(a)** Comparison of cross-frame tracking accuracy in different scenarios; **(b)** Changes in target position error across consecutive frames.

Figure 4a shows that all models maintain high tracking accuracy in unoccluded scenarios, with the proposed method reaching 95.3%, an improvement of 2.5 percentage points compared to VideoMAE. This indicates that predictive attention provides a stable gain for target state estimation under normal motion conditions. After target occlusion occurs, the accuracy of all models decreases, but the proposed method still maintains 91.8%, an improvement of 8.1 percentage points compared to VideoMAE and 13.2 percentage points compared to TASED-Net. Under camera motion conditions, it further reaches 89.6%, an improvement of 8.2 percentage points compared to VideoMAE, indicating that the predictive state constraint weakens the target drift accumulation caused by complex motion. Figure 4b further reflects the change in target position error across consecutive frames. After the occlusion begins, the VideoMAE error rapidly increases from 5.5 pixels to 15.3 pixels, TASED-Net increases to 18.5 pixels, while the proposed method only increases to 8.4 pixels. The peak error during the camera movement phase reaches 17.5 pixels, 20.7 pixels, and 9.6 pixels, respectively, and then gradually decreases to 10.8 pixels, 12.7 pixels, and 6.2 pixels. This indicates that the predictive attention module maintains the continuity of target state estimation, reduces target temporal drift, and achieves more stable cross-frame tracking performance in complex film and television scenes.

### 4.3 Scene semantic classification performance analysis

Semantic understanding of film and television scenes involves various scene types, including static environments, character interactions, and dynamic events. Significant differences exist between different categories in appearance features, motion patterns, and semantic relationships. Therefore, the MovieNet dataset was used to evaluate the model’s scene-level semantic representation capabilities. Experiments were conducted around eight typical film and television scene categories: Indoor, Outdoor, Dialogue, Action, Crowd, Vehicle, Night, and Transition. Indoor and Outdoor correspond to indoor and outdoor environments, respectively; Dialogue reflects character dialogue scenes; Action corresponds to violent movement events; Crowd represents scenes of multiple people gathering; Vehicle describes transportation-related scenes; Night represents nighttime shooting environments; and Transition corresponds to shot changes and scene transitions. The methods were compared under the same training strategy and testing conditions. Average Precision (AP) was used to measure the single-class recognition performance, and the semantic discriminative characteristics of the model were analyzed in conjunction with the inter-class prediction relationships. The results are shown in Figure 5.

**Figure 5:**
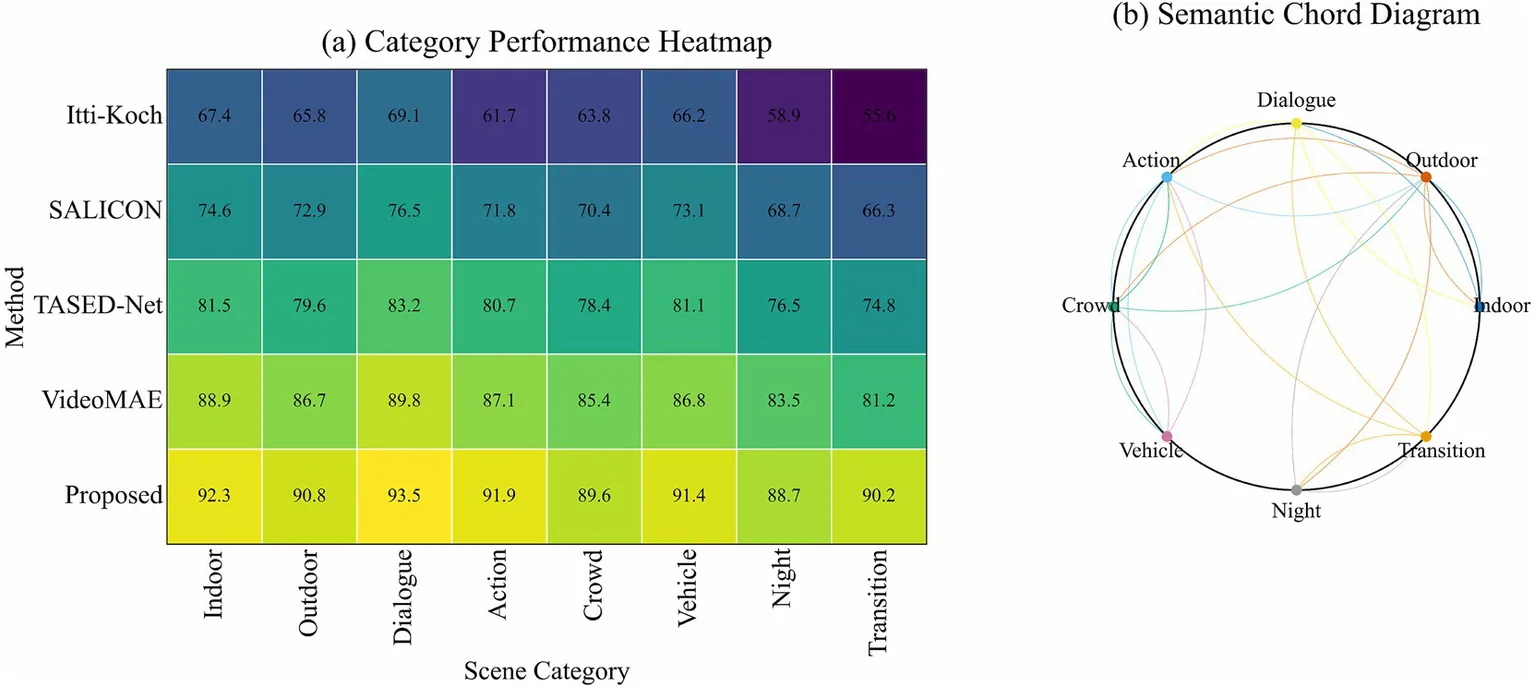
Analysis of MovieNet scene semantic classification results **(a)** Category Performance Heatmap; **(b)** Semantic Chord Diagram.

Figure 5a shows the classification performance distribution of different models across various film and television scenes. The traditional Itti-Koch model has an overall AP ranging from 55.6% to 69.1%, which is insufficient for high-level semantic representation in complex film and television scenes. After introducing deep feature learning, SALICON and TASED-Net improved the AP of each category to 66.3%–76.5% and 74.8%–83.2%, respectively. VideoMAE further improved the AP to 81.2–89.8% by relying on video pre-training. The proposed method achieved the highest performance in all eight scene categories: Dialogue (93.5%), Indoor (92.3%), Action (91.9%), Vehicle (91.4%), Night (88.7%), Transition (90.2%), Outdoor (90.8%), and Crowd (89.6%). The performance fluctuations between categories were significantly reduced, indicating that the dual-stream spatiotemporal joint representation enhances the discrimination ability of dynamic scenes and complex lighting conditions while maintaining the semantic consistency of static scenes, enabling different scene categories to obtain more balanced semantic representations. Figure 5b further illustrates the predictive relationships between scene categories. Diagonal region connections dominate, indicating that most samples maintain correct classification. There are still a few cross connections between Outdoor and Night, Action and Crowd, and Dialogue and Indoor, reflecting the local overlap of visual features in semantically similar scenes. However, the overall number of mismappings is small, and the degree of confusion between categories is effectively suppressed. This shows that predictive attention and cross-frame semantic aggregation together enhance the semantic discrimination ability of complex film and television scenes.

### 4.4 Ablation analysis of predictive attention module

To analyze the impact of predictive attention mechanisms and related key modules on overall performance, DS-STEN was modularly ablated while maintaining consistency in dataset partitioning, training strategies, hyperparameter configurations, and optimization processes. The complete model is denoted as M0. M1 removes Kalman prediction and predictive attention mask injection, retaining only the original spatiotemporal features for subsequent semantic modeling. M2 replaces the Transformer cross-frame semantic aggregation module with global average pooling and fully connected layers. M3 retains the network structure and uses only saliency prediction loss for single-task training. M4 retains Kalman state prediction but removes the predictive attention mask injection mechanism to distinguish the independent roles of state prediction and prediction-guided feature modulation. Each model was trained five times, and the mean and standard deviation were calculated. CC was used to evaluate saliency prediction consistency, Tracking Accuracy and IoU were used to measure cross-frame target preservation and localization accuracy, and mAP was used to evaluate scene semantic classification performance. Experimental results are shown in Table 3.

**Table 3:** Experimental results of ablation of the predictive attention module.

Model	Kalman prediction	Prediction attention mask	Transformer	Multi-task learning	CC ↑	Tracking acc. (%) ↑	IoU ↑	mAP (%) ↑
M0	✓	✓	✓	✓	0.846 ± 0.005	78.9 ± 0.6	0.721 ± 0.006	61.8 ± 0.5
M1	✕	✕	✓	✓	0.814 ± 0.008	73.5 ± 0.9	0.667 ± 0.010	58.4 ± 0.8
M2	✓	✓	✕	✓	0.828 ± 0.006	75.6 ± 0.8	0.691 ± 0.008	56.7 ± 0.7
M3	✓	✓	✓	✕	0.835 ± 0.006	76.8 ± 0.7	0.703 ± 0.007	59.2 ± 0.6
M4	✓	✕	✓	✓	0.822 ± 0.007	74.7 ± 0.8	0.682 ± 0.009	59.6 ± 0.7

The complete model achieved optimal results across all metrics, with CC at 0.846 ± 0.005, Tracking Accuracy at 78.9% ± 0.6%, IoU at 0.721 ± 0.006, and mAP at 61.8% ± 0.5%. After removing the Kalman prediction and prediction attention mask, Tracking Accuracy decreased to 73.5%, IoU to 0.667, and CC to 0.814, indicating a significant weakening of cross-frame target anchoring ability and a simultaneous decrease in saliency response stability due to missing prediction priors. Removing the Transformer reduced mAP to 56.7%, a larger decrease than other variants, reflecting the crucial role of cross-frame global semantic aggregation in understanding film and television scenes. Single-task training reduced CC, Tracking Accuracy, and mAP to 0.835, 76.8%, and 59.2%, respectively, demonstrating that joint supervision enhanced the discriminative ability of shared features. When only the prediction attention mask is removed, the tracking accuracy drops to 74.7% and the IoU drops to 0.682, but the overall performance is still better than completely removing the prediction module. This indicates that state prediction and prediction attention injection together constitute a cross-frame feature correction mechanism, and their synergistic effect supports the model’s stable representation ability.

### 4.5 Robustness analysis of semantic understanding in complex film and television scenarios

To evaluate the semantic preservation capability of the proposed model in complex film and television scenes, progressively complex scenes were constructed using typical film and television conditions such as dense montage editing, sudden changes in lighting, and their superposition. These scenes were then divided into six test states based on their complexity. P1 corresponds to a normal scene, P2 to light montage editing, P3 to dense montage editing, P4 to sudden changes in lighting, P5 to the combined effect of montage editing and sudden changes in lighting, and P6 to an extremely complex film and television scene. The experiment used Scene-level Semantic Consistency and Event-level Semantic Consistency to measure the consistency of scene-level and event-level semantics in continuous video, respectively. The stability of the model output was evaluated from two levels: overall scene understanding and event representation. The results are shown in Figures 6, 7.

**Figure 6:**
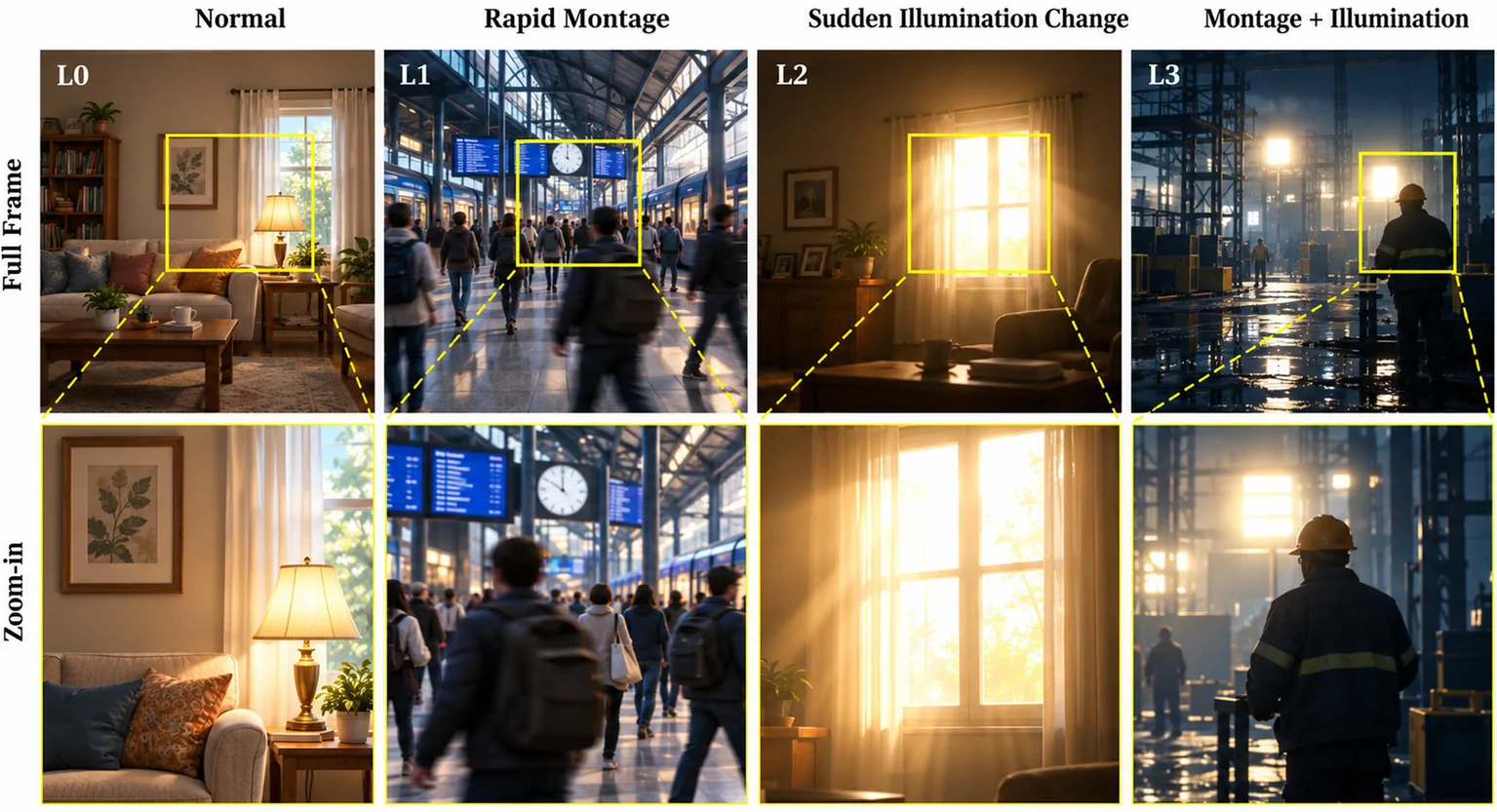
Examples of typical complex scenes in film and television. AI-assisted illustrative elements generated using OpenAI ChatGPT Images; see Supplementary material for prompts, tool details, and disclosure. The illustrative scenes are conceptual, not actual experimental video frames.

**Figure 7:**
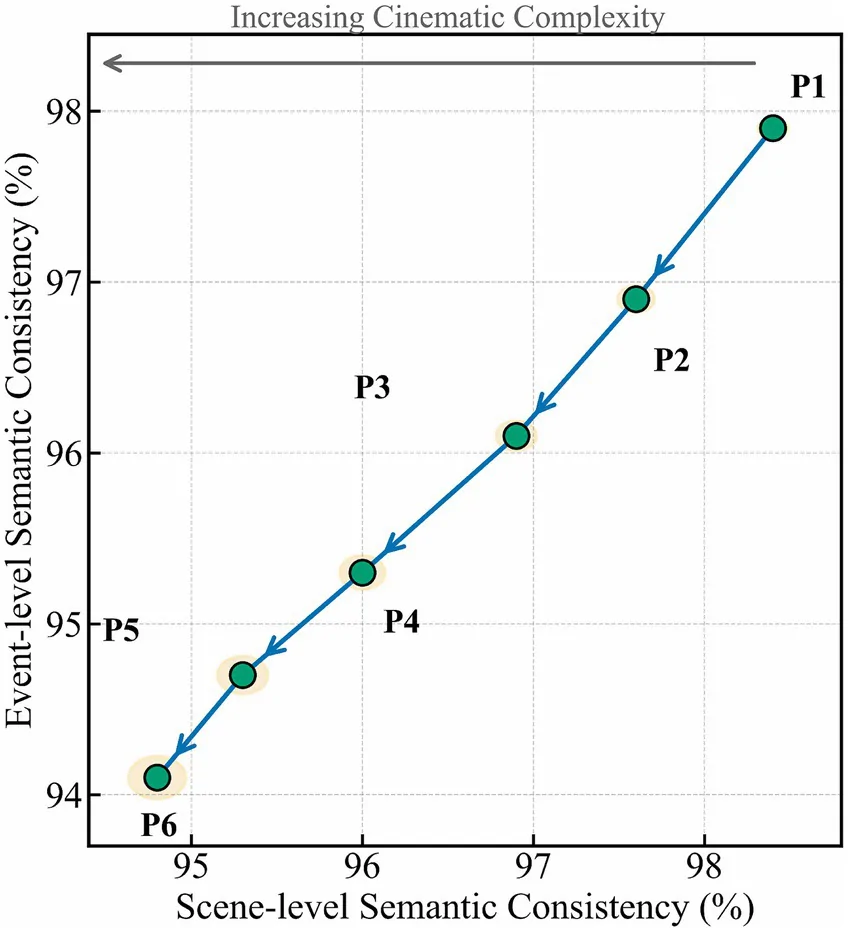
Semantic output stability trajectory under different film and television complexity conditions.

Figure 6 illustrates the gradual increase in complexity of film and television scenes from normal to extreme conditions. The visual continuity weakens as the scene undergoes increased editing frequency, drastic changes in lighting, and interference from multiple coupled factors, leading to a gradual degradation of local details in the target area and posing greater challenges to scene-level and event-level semantic understanding. Figure 7 shows that as the complexity of film and television increases, the model’s semantic output trajectory remains relatively stable. Scene-level Semantic Consistency decreases from 98.4% to 94.8%, a cumulative decrease of 3.6%, while Event-level Semantic Consistency decreases from 97.9% to 94.1%, a cumulative decrease of 3.8%. Scene-level semantic consistency is consistently higher than event-level semantic consistency, indicating that scene semantics has stronger global stability, while event semantics is more sensitive to rapid shot transitions and lighting disturbances. Both metrics remain above 94%, with no abrupt changes or significant shifts in the trajectory, demonstrating that the proposed method maintains stable scene-level and event-level semantic representations under complex film and television conditions and exhibits strong adaptability to the specific complexities of film and television.

## 5 Conclusion

This paper addresses the temporal drift of semantically salient targets and the instability of cross-frame semantic associations in complex film and television videos. A unified framework integrating brain-like visual attention, spatiotemporal joint representation, and predictive semantic modeling is constructed. Experimental results show that physiological-level visual attention constraints, predictive state estimation, and cross-frame context aggregation form an effective synergy, improving the continuity, stability, and discriminative ability of semantic representations under complex editing, target occlusion, and illumination changes, validating the modeling advantages of brain-like prediction mechanisms in understanding complex film and television videos. However, limited by the linear state prediction assumption and fixed temporal modeling method, the model still has room for improvement in generalizing to long-term narrative dependencies, multi-target interaction scenarios, and cross-data domains. This study provides a new approach for the deep integration of neuroscience predictive coding and film and television video semantic understanding, enriching the theoretical research on brain-like visual computing in modeling complex dynamic scenes. Future research will further explore nonlinear dynamic prediction, adaptive long-term memory, and multimodal semantic collaborative modeling to continuously improve the expressive power and generalization performance of brain-like visual semantic understanding in complex film and television scenes.

## Data Availability

The original contributions presented in the study are included in the article/Supplementary material, further inquiries can be directed to the corresponding author.
