# Supplementary material for: Brain-inspired predictive visual attention for spatiotemporal semantic understanding of complex film and television videos
Source: Front Neurorobot. 2026 Oct 22;20:1934032. doi: 10.3389/fnbot.2026.1934032 (PMC13735105; doi:10.3389/fnbot.2026.1934032)
Supplement: Supplementary file 1 [file Supplementary_file_1.docx]

Supplementary Material: Generative AI Disclosure for Revised Figures 1 and 6

**Article:** Brain-inspired predictive visual attention for spatiotemporal semantic understanding of complex film and television videos

**Author:** Peng Han	**DOI:** 10.3389/fnbot.2026.1934032

**Purpose.** This disclosure documents the use of a generative AI image tool to prepare replacement conceptual illustrations for two figures during the production-stage copyright clarification. It reports the tool identity, publicly documented release family, source, image-generation instructions, image inputs, and intended scope of use.

# 1. Tool identification and version information

| Provider | OpenAI |
| --- | --- |
| Tool / product | ChatGPT Images (image generation and editing within ChatGPT) |
| Public product version | ChatGPT Images 2.5 was the publicly documented ChatGPT image product release in September–October 2026. This does not establish the exact internal model build used for these specific outputs. |
| Exact model/build identifier | Not exposed in the available ChatGPT image-generation record or image file metadata; therefore not asserted. |
| Software URL | https://chatgpt.com/ |
| Product documentation | https://help.openai.com/en/articles/11084440-images-in-chatgpt |
| Release notes | https://help.openai.com/en/articles/6825453-chatgpt-release-notes |
| Method | AI-assisted editing/generation using the previously prepared manuscript figure images as layout references, with instructions to replace third-party scene imagery with new conceptual illustrations. |

# 2. Scope, inputs, and safeguards

- Original Figure 1 (network architecture diagram) and original Figure 6 (four-scenario comparison) were provided as image references to preserve the arrangement and intended comparisons.
- For Figure 1, the replacement mainly concerns the city photographs in the RGB Frames strip; the network architecture and technical labels were instructed to remain unchanged.
- For Figure 6, the previous scene photographs were replaced with AI-generated illustrative scenes. The generated panels are conceptual visualizations and must not be represented as actual sampled frames, validation images, or measured experimental outputs.
- Neither replacement figure was used to collect experimental data, train or validate the reported model, compute metrics, produce statistical analyses, or derive scientific conclusions. Authors remain responsible for checking labels, technical accuracy, source rights, and consistency with the final manuscript before approval.

# 3. Figure 1: generation record

**Output file:** spatio_temporal_video_representation_pipeline.png | 1774 × 887 pixels

**Generation record ID:** f12cd8f5-ab7c-49ab-a53e-7cf3bf8e832f

*Prompt supplied to the image-generation workflow (verbatim editorial instruction):*

Edit Image A to create a publication-ready replacement for Figure 1 while preserving the overall academic diagram layout, module structure, labels, arrows, dimensions, and color coding. Image A is the direct edit target. Image B is supplemental context only and should not be copied. Keep the figure as a clean scientific schematic. Preserve the three-column layout: Spatial Stream on the left, Saliency Heatmap in the center, and Temporal Stream on the right, along with the fusion pathway at the bottom. Keep the existing text labels and the architecture content essentially unchanged, including: 'RGB Frames', 'Saliency Heatmap', 'Optical Flow Sequence', 'Resize(bilinear)', 'Attention Weight (7x7)', 'Enhanced Spatial Feature F_s', 'ResNet-50 (Spatial Encoder)', 'Clip-aware Temporal Windowing (Shot Boundary Guided)', '3D Convolutional Block (Temporal Encoder)', 'Temporal Feature F_t', 'Channel Concatenation (Along Channels)', '1 × 1 Convolution', and 'Unified Spatio-temporal Representation F'. Also preserve the dimensional annotations such as T, 224 × 224 × 3, 224 × 224, 224 × 224 × 2, 56 × 56 × 256, 28 × 28 × 512, 14 × 14 × 1024, 7 × 7 × 2048, and the fused tensor dimensions. The critical change: replace the real city photographs in the 'RGB Frames' strip with original, author-created illustrative video frames that are clearly schematic and non-copyrighted. Use simple generic urban/coastal silhouette scenes or abstract cinematic scene frames, designed as stylized frames inside filmstrip borders, not copied photos. Ensure the saliency heatmap thumbnails and optical flow thumbnails remain coherent and schematic. Maintain high readability, neat typography, crisp lines, a light neutral background, and the original paper-figure aesthetic.

*Generated Figure 1 — preview*

# 4. Figure 6: generation record

**Output file:** lighting_and_montage_comparison_chart.png | 1672 × 941 pixels

**Generation record ID:** 9c074fa1-0478-4e12-b1d5-da9df4f08a72

*Prompt supplied to the image-generation workflow (verbatim editorial instruction):*

Edit Image B to create a publication-ready replacement for Figure 6 while preserving the same comparative layout and labels. Image B is the direct edit target. Image A is supplemental context only and should not be copied. Keep the four columns with headings 'Normal', 'Rapid Montage', 'Sudden Illumination Change', and 'Montage + Illumination'. Keep the left-side row labels 'Full Frame' and 'Zoom-in'. Keep the yellow focus rectangles in the top row and the dashed yellow connector lines leading to the enlarged bottom-row images. Keep labels L0, L1, L2, and L3 in the top images. The critical change: replace all real photo/film frames with original, non-copyrighted concept illustrations that communicate the same four visual conditions. Use a polished semi-realistic digital illustration style suitable for an academic figure, not photojournalistic realism and not copied film stills. Column 1 (Normal): a cozy living room interior with sofa, side table, lamp, and window, evenly lit. Column 2 (Rapid Montage): a busy transit-hall or station-like public interior with moving people and screens, conveying rapid visual change. Column 3 (Sudden Illumination Change): a dim interior dominated by a suddenly bright glowing window with strong backlight bloom. Column 4 (Montage + Illumination): a dark industrial or warehouse-like scene with a silhouetted worker-like figure and multiple bright backlights, combining complex composition and illumination changes. The bottom row should show zoomed-in crops corresponding to the highlighted top-row areas. Make the composition clean, balanced, and clearly original, with a white background and strong readability for publication.

*Generated Figure 6 — preview*
